# Interantral alveolar ridge splitting for maxillary horizontal expansion and simultaneous dental implant insertion: A case report

**DOI:** 10.1016/j.amsu.2019.10.018

**Published:** 2019-10-28

**Authors:** Sebastian Berger, Paul Hakl, Walter Sutter, Marius Meier, Henning Roland, Patrick Bandura, Dritan Turhani

**Affiliations:** a Centre for Oral and Maxillofacial Surgery, University of Dental Medicine and Oral Health, Danube Private University (DPU), Krems, Austria; b Private Surgery, Maria Enzersdorf, Austria

**Keywords:** Case report, Alveolar ridge splitting technique (ARST), Interantral region, Full arch rehabilitation, Narrow alveolar ridge, Implant-retained rehabilitation, ARST, alveolar ridge splitting and augmentation technique, CBCT, Cone-Beam Computed Topography, GBR, guided bone regeneration

## Abstract

**Introduction:**

Dental implants present an advanced technique for the rehabilitation of partial or edentulous patients. Tooth loss caused by caries, periodontal disease or systemic factors often results in a decline of the bucco-lingual alveolar ridge dimension. Within one year the initial bone width can be resorbed up to 50%. As a consequence dental implants may be limited for rehabilitation and cannot be performed in a conventional manner because of the risk of dehiscence and fenestrations. Bone blocks, guided bone regeneration (GBR), horizontal osteogenic distraction and bone grafts may be used for augmentation procedures. In case of sufficient vertical bone dimension, an alveolar ridge splitting and augmentation technique (ARST) can be conducted. This case has been reported in line with PROCESS criteria [1].

**Case presentation:**

We present a 51-year old female patient, who has had a full denture for about 30 years. The reason for consultation was the demand for a fixed prosthesis. Dental implants in combination of the ARST with GBR allowed us to correct horizontal deformities of the alveolar ridge.

**Discussion:**

We discuss the possibility of using the ARST in the interantral region for a full arch rehabilitation of the maxilla with simultaneous dental implant placement in a narrow alveolar ridge.

**Conclusion:**

The ARST in addition to simultaneous implant placement with a GBR can be successfully used for a full arch rehabilitation of the maxilla in a narrow alveolar ridge.

## Highlights

• ARST can be successfully used for a full arch rehabilitation of the maxilla in a narrow alveolar ridge.
• ARST resulted a horizontal bone width gain of 3 mm.
• ARST and mini implants show a stable result over 4 years.
• Dental implants in combination of the ARST with GBR can correct horizontal deformities of the alveolar ridge.
• Second stage surgeries like GBR, autogenous block onlay grafts, or distraction osteogenesis can be avoided entirely, by ARST.

## 1 Introduction

Tooth loss is associated with adverse effects in terms of general health and social interaction. The number of edentulous patients is increasing as the elderly population increases. Current evidence suggests that implant-retained prostheses result in greater increases in patient satisfaction and oral health-related quality of life compared to conventional dentures [2]. Nevertheless, in the event of early tooth loss, the buccolingual alveolar ridge dimension decreases significantly which poses difficulties for implant placement [3,4].

In the past two decades, several surgical techniques have been established to manage an extremely atrophic maxillary alveolar ridge. The alveolar ridge-splitting/expansion technique (ARST), with or without guided bone regeneration (GBR) during implantation, has become an established method for horizontal bone augmentation [3,5,6]. In recent years, several ridge-splitting techniques have been developed, including split crest osteotomy, ridge expansion osteotomy, and various other modifications [5]. A wide array of tools for splitting have also been developed, including hammers, spatulas, motorized ridge expanders, and rotating or oscillating saws [3,5,7].

A successful implantation using ARST requires a minimum alveolar bone width of 3 mm to ensure sufficient trabecular bone substrate, as well as cortical and cancellous bone on both sides of the split ridge [3,5]. This minimum width is also necessary for bone spreading purposes and for maintaining a suitable blood supply to the bone adjacent to the implant [3,5]. In addition, ARST requires the absence of concavity in the alveolar bone profile, and a minimum vertical bone height of 10 mm [3]. ARST has several advantages such as the possibility of simultaneous implant placement; avoiding the need for secondary donor sites for bone graft harvesting; reducing morbidity; and shortening of the treatment time [8].

In most of the approaches used for the rehabilitation of an edentulous maxilla with implants, 4 to 6 implants have to be placed in the interantral area [[9], [10], [11]]. The interantral alveolar bone borders distally on variously pneumatized maxillary sinuses and contains the canine and incisor area in 96.9% of individuals [8]. Generally, two implants are placed symmetrically in the incisor areas, and two additional implants are placed bilaterally in the canine and/or premolar areas [9,10]. This report presents a case in which the edentulous maxilla was rehabilitated with an implant-supported full-arch fixed prosthesis. We report the present case in accordance with the SCARE criteria [1].

## 2 Presentation of case

**Fig. 1:**
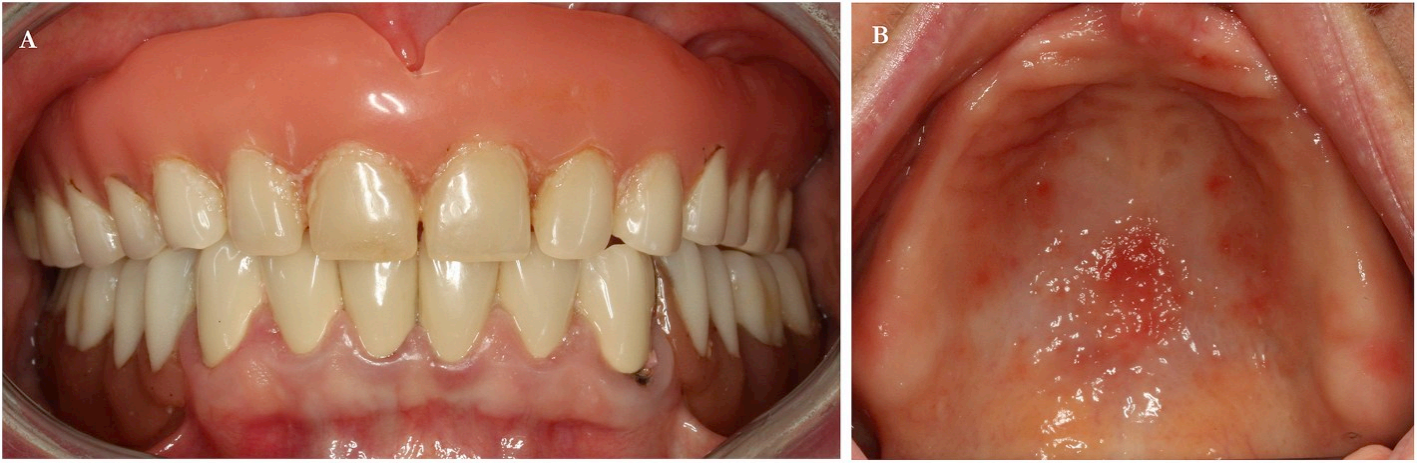
*A,* Intraoral initial situation demonstrating full denture in the upper jaw. *B,* Palatal view of the pre-surgical situation.

In May 2014, a 51-year-old female patient presented with complete maxillary edentulism at our institution. Her chief complaint was the request for a fixed prosthesis. Other than the use of antithyroid medication for a non-contributory but symptom-free hyperthyroidism, her medical history was otherwise unremarkable. Since her pregnancy 30 years ago, she has had a maxillary complete denture (Fig. 1a–b). Cone-beam computed topography revealed an adequate bone height but insufficient bone width that was reduced to a minimum of 2 mm.

**Fig. 2:**
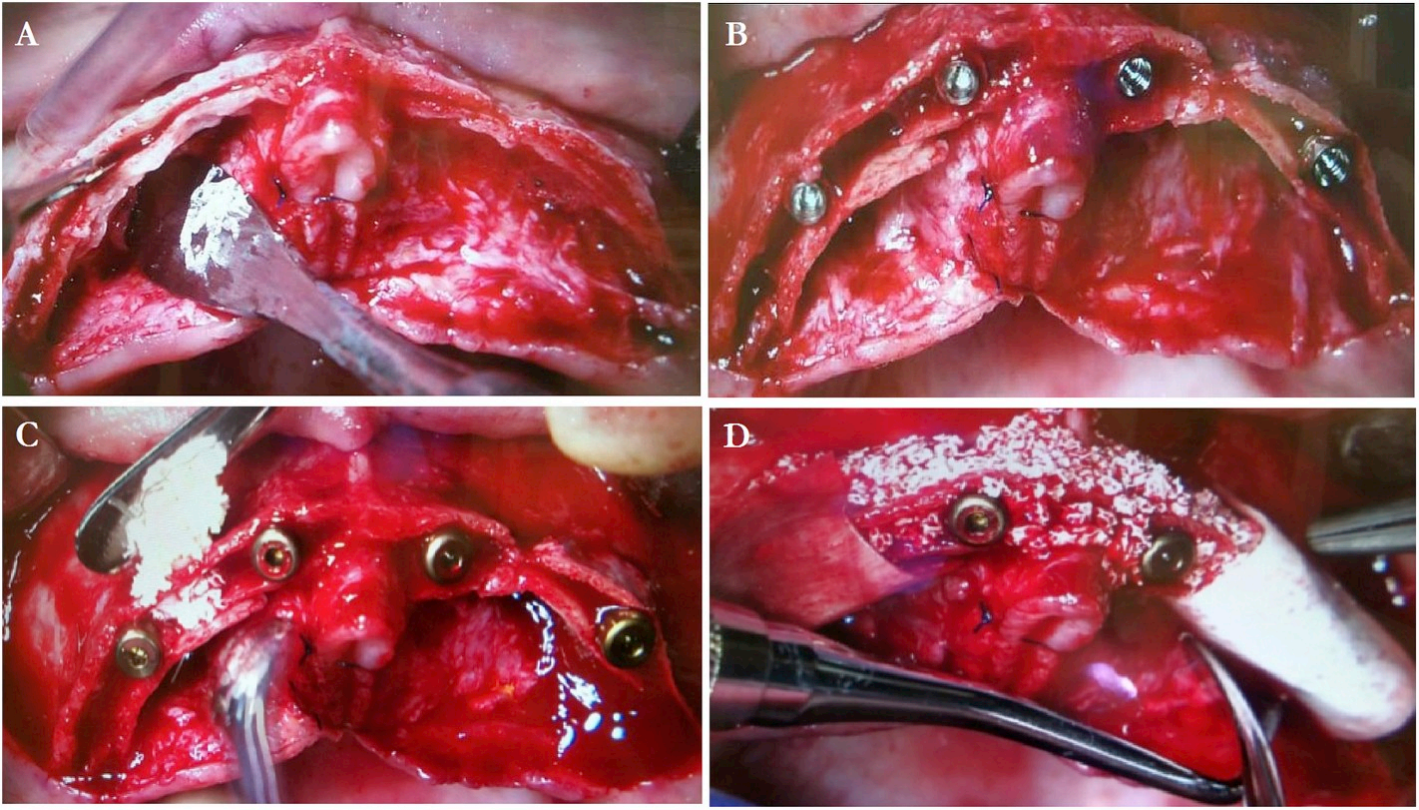
*A,* midcrest osteotomy of the alveolar bone. *B,* immediate implant treatment with four mini implants after ARST. *C + D,* guided bone regeneration using a mixture of autologous bone chips and a xenogeneic bone replacement and a collagen membrane.

In January 2015, she underwent surgery under local anesthesia performed by our department head. A full thickness mucoperiosteal incision flap was made. The alveolar ridge was then smoothed out, and bone chips were simultaneously harvested (Fig. 2a-d). A midcrest osteotomy of the alveolar bone, without vertical cuts, was performed using a piezoelectric device (Piezotome 2 with CS1 attachment, Acteon Germany GmbH, Industriestraβe 9, D-40822 Mettmann, Germany) (Fig. 2a). Osteotomes were used to widen and split the alveolar bone into the regions of the right and left first premolars (Osteotom Bone-Spreader #3 convex, maxi 3,5 mm Hu-Friedy Mfg. Co LLC, 60528 Frankfurt am Main, Germany). In the region of the left first premolar, slight cracks in the buccal wall occurred.

Thereafter, an immediate implantation with four mini implants was performed (three 2,7 × 11,5 mm and one 2,9 × 11,5 mm Mini Bego Semados, Bego Implant Systems GmbH & Co KG, 28359 Bremen, Germany) (Fig. 2b). All implants were installed with a programmed motor torque control and/or ratchet/torque spanner set at 20 N or more, and maintained with sealing caps. GBR was performed using a mixture of autologous bone chips and xenogeneic bone replacement material (Endobon Xenogen R Granules, Biomet3i Deutschland GmbH, Warsaw US- Indiana), and a collagen membrane (Bego Collagen Membrane 15 × 20 mm, Bego Implant Systems GmbH & Co KG, 28359 Bremen, Germany) (Fig. 2c and d). The soft tissue was sutured back with 4-0 nonresorbable sutures (Hu-Friedy Mfg. Co. LCC, Frankfurt).

**Fig. 3:**
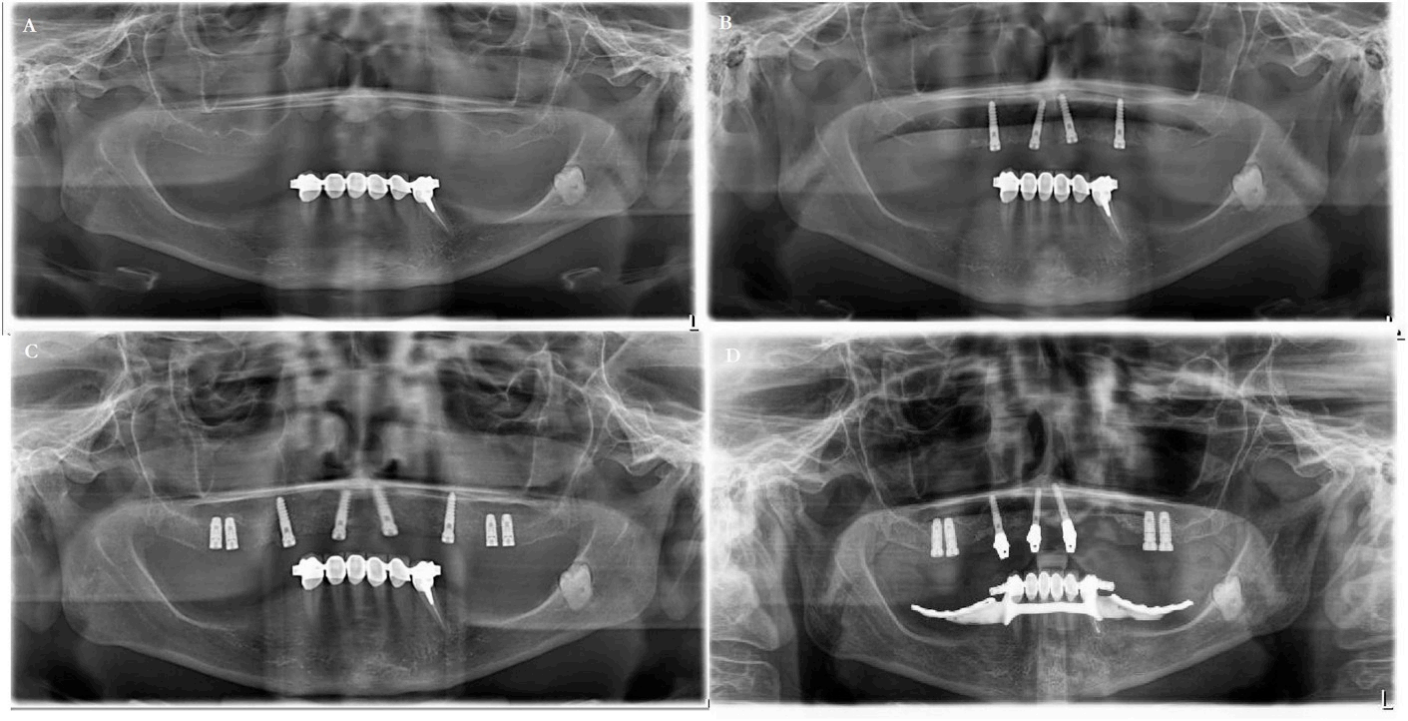
*A,* re-surgical panoramic radiographic image (2014). *B,* Panoramic radiographic image demonstrating situation after first surgery (immediate implant treatment with four mini implants was performed ARST (2015). *C,* Panoramic radiographic image demonstrating situation after second surgery of implant placing in the molar region (2015). *D,* Panoramic radiographic image demonstrating in 2019.

Eight weeks later, a second surgery was carried out, and implants were placed in the molar region. Internal sinus lifts were performed using appropriate osteotomes of increasing diameter to compress the surrounding bone (Osteotom Bone-Spreader #3 convex, maxi 3,5 mm Hu-Friedy Mfg. Co LLC, 60528 Frankfurt am Main, Germany). Drills were not used as the bone was observed to be soft and of poor quality. The bone was merely widened, and four implants of 3.75 × 8.5 mm sequence took place. The bone was merely widened, and four implants of 3.75 × 8.5 mm (Bego Semados S3,75 L 8,5; Bego Implant Systems GmbH & Co KG, Bremen, Germany) were placed with a torque of 35 Ncm, and maintained with sealing caps (Fig. 3a–d and Table 1).

**Fig. 4:**
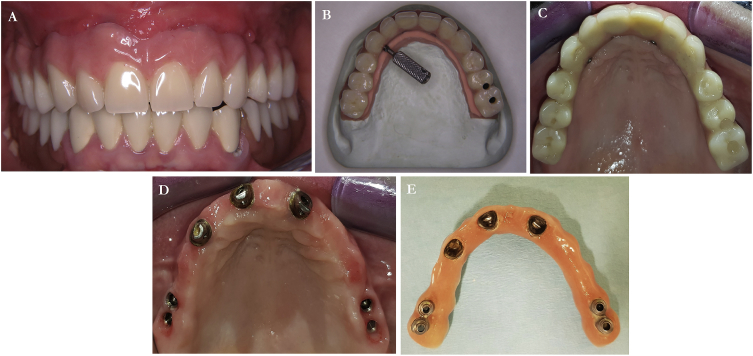
*A,* Prosthodontic rehabilitation frontal view. *B,* palatal view before insertion. *C,* palatal view in-situ. *D,* palatal view without restauration. *E,* restauration outside the mouth after four years.

**Table 1:** Presents the pre-surgical horizontal bone width, the horizontal bone gain after ARTS and after six months.

time/region	region 12	region 14	region 22	region 24
Pre-surgical	2,22 mm	2,55 mm	1,89 mm	2,09 mm
after ARST	5,52 mm	5,38 mm	6,09 mm	5,12 mm
after 6 months	5,48 mm	5,04 mm	5,22 mm	5,49 mm

After 7 months of healing, all but one implant in the region of the first left premolar were osseointegrated very well, and the healing period was free of complications and an implant-supported prosthesis was delivered. In the anterior area, the prosthesis was fixed on four metal abutments with palatal screws, whereas in the posterior region, the prosthesis was screwed directly to the implants (Fig. 4).

## 3 Discussion

A patient's request for a fixed prosthesis on an atrophic and narrow edentulous ridge presents as a challenge for prosthodontists and oral surgeons. In the present case, there was severe horizontal bone loss. Before the intervention, the patient was informed in detail about the advantages and disadvantages of interantral alveolar ridge splitting for maxillary horizontal expansion. The patient was also informed of alternatives including the fabrication of a new conventional complete denture, a prosthesis attached to at least two locators, or an implant-retained bridge.

The alveolar ridge expansion technique by Tatum et al. was first described in 1986, and the ARST by Simion et al. was essentially developed to achieve a more conservative surgical procedure [12]. Second-stage surgeries such as GBR techniques, autogenous block onlay grafts, or distraction osteogenesis can be avoided entirely, along with all their disadvantages [3,8]. In contrast, technical complications using the ARST are low at approximately 6.8% [13].

Certain prerequisites must be attained for ARST, such as a minimum alveolar bone width of 3 mm or more, and a vertical dimension of 10 mm with no pronounced concavity [3]. However, in cases where the bone width is less than 3 mm, the risk of fractures increases since the partition is almost exclusively made in the cortical bone [14]. Ridges should expose the bone marrow between the buccal and lingual/palatal walls [14]. With a sufficient residual horizontal bone dimension, ARST has a high clinical success rate when performed for maxillary arches [15]. A previous meta-analysis reported a horizontal bone width gain of approximately 4.13 ± 3.13 mm [8].

With D3 and D4 bone quality, the bone is pliable and can be compacted [15]. While there is a lack of long-term studies, preliminary evidence suggests that the success rates for implant placement using ARST in cases with narrow arches are comparable to those in pristine bone but with a slightly increased buccal bone loss [3,8]. In addition to the introduction of the microsaw or piezoelectric devices, precise cutting is possible, which greatly simplifies the split crest procedure [7,16]. The ultrasonic frequency does not cut soft tissue and consequently causes less collateral tissue damage [17]. An important aspect in atraumatic preparation is to secure the periosteum, especially on the buccal side, as it serves as a protective membrane that heals microfractures and ensures an adequate blood supply [15]. The reported survival rates of titanium implants using ARST were approximately 96% at 58 months [6]. In the present case, we used bovine bone substitution material and a pericardium membrane to close the gaps between the implants. Although biomaterial grafts are not necessary for osseointegration, they contribute to a better contour [14]. Blus and Szmukler-Moncler described the use of an ultrasonic device, in addition to GBR and xenogetic bone replacement material, as an effective combination for implant placement with a success rate of approximately 96.5% [18].

Nonetheless, ARST has several disadvantages. It is an operator-dependent technique with a learning curve [16], and the surgeon should operate at least ten bone splitting cases per year, so that this kind of case can be operated successfully. The benefits of ARST are limited to horizontal bone gain [16], and it should be used preferably only for D2, D3, and D4 bone types [15]. Padmanabhan and Gupta reported a greater crestal bone loss with the ridge expansion technique developed by Summer, which involved the use of osteotomes [15]. Single tooth areas, as opposed to an entire ridge, present with greater challenges when using ARST because of the lack of bone elasticity [15]. ARST, however, is not without complications, which may include implant loss, fractures in the medullar bone tissue between the two cortical plates due to the presence of risks including weakened anatomical conditions of the narrow jaw comb, as well as clinical infections caused by wearing the prosthesis during the transitional period or by inadequate adaptation of the mucous membrane lobe [19,20].

## 4 Conclusion

**Fig. 5:**
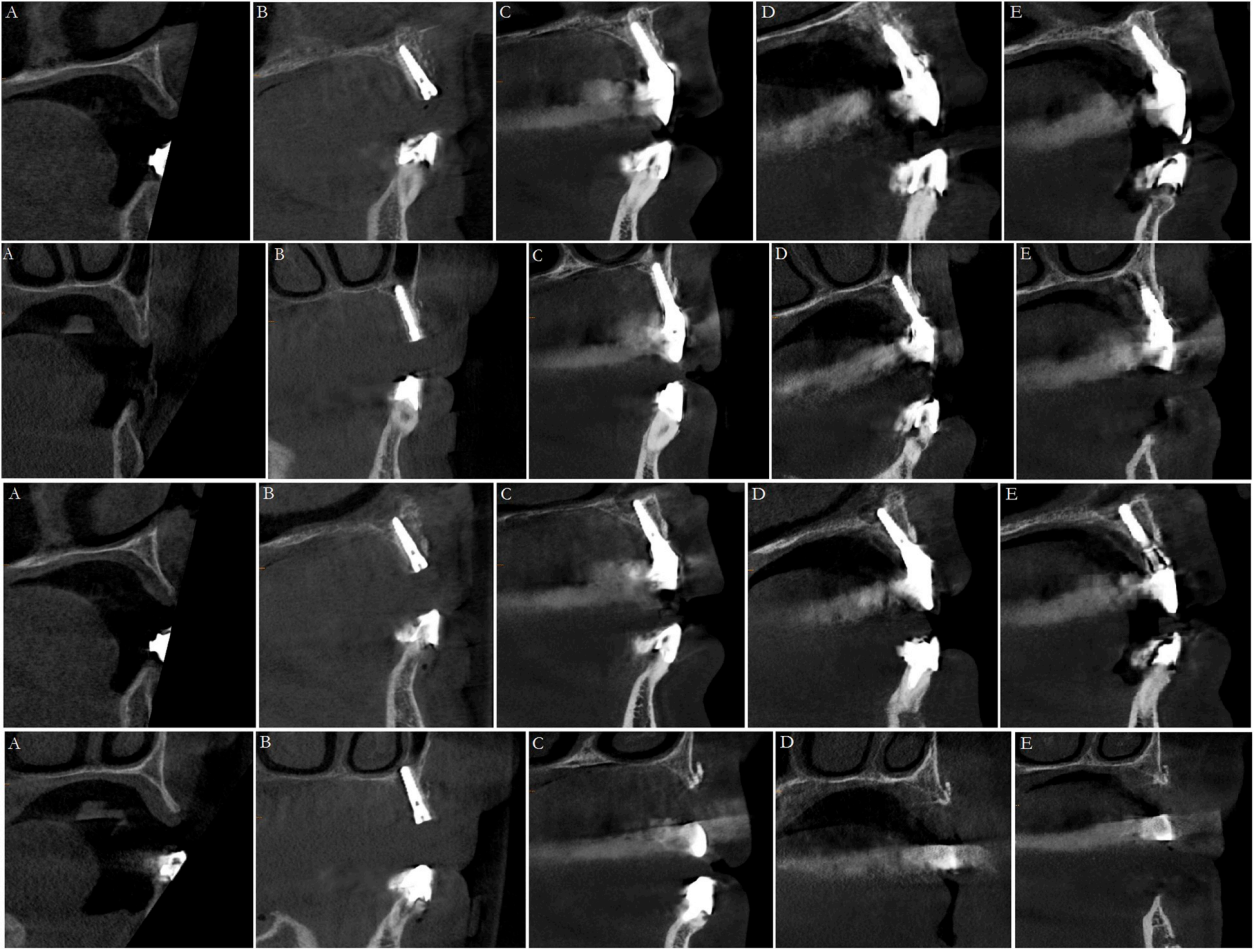
*A-E,* Cone beam computed tomographic (CBCT) imaging, comparing alveolar ridge with before and after placing of implants in region 12, 14, 22, 24 *A,* Pre-surgical status revealing moderately deficient alveolar ridge dimension of the maxillary ridge 2015. *B,* CBCT after surgery using ARST and dental implant placement; note that the alveolar ridge was sufficiently expanded. *C,* CBCT 2017. *D,* CBCT 2018. *E,* CBCT 2019.

As this case shows, stable results may be achieved using ARST up to a period of at least 4-years (Fig. 5a to e). In order to ensure the success of a fixed prosthesis, patient compliance is paramount, especially with regards to regular care and long-term maintenance. Although additional studies with large sample sizes and longer-term follow-up periods are required to confirm the effectiveness of ARST using a full-arch alveolar ridge split, our preliminary results suggest that this technique may be suitable for the management of edentulous patients with limited horizontal bone width.

## Ethical approval

Not applicable. The present study is not a research study.

## Sources of funding

This research received no specific grant from any funding agency in the public, commercial, or not-for-profit sectors.

## Author contribution

Paul Hackl and Sebastian Berger provided clinical care to the patient, wrote themanuscript, and coordinated all aspects of this report.

Dritan Turhani performed the surgery on the patient and provided clinical, radiological, and photographic documentation.

Walter Sutter, Marius Meier, Henning Roland and Patrick Bandura performed clinical and radiological follow-up treatment.

All authors read and approved the final manuscript.

## Research registration number

Not applicable. No research study involved.

## Guarantor

Dritan Turhani, Paul Hakl and Sebastian Berger.

## Consent

The patient received a thorough explanation of this report gave her oral and written informed consent to be included in this report as well as for publication of these case, anonymous data, and pictures. A copy of the written consent is available for review on request.

## Provenance and peer review

Not commissioned, externally peer reviewed.

## Declaration of competing interest

The authors declare that there is no conflict of interest.

## Appendix A Supplementary data

SCARE_Checklist SCARE_Checklist
